# Harnessing the Potential of NK Cell-Based Immunotherapies against Multiple Myeloma

**DOI:** 10.3390/cells11030392

**Published:** 2022-01-24

**Authors:** Chantal Reina-Ortiz, David Giraldos, Gemma Azaceta, Luis Palomera, Isabel Marzo, Javier Naval, Martín Villalba, Alberto Anel

**Affiliations:** 1 Apoptosis, Immunity & Cancer Group, Department Biochemistry and Molecular and Cell Biology, Faculty of Sciences, University of Zaragoza and Aragón Health Research Institute (IIS Aragón), 50009 Zaragoza, Spain; giraldeux@gmail.com (D.G.); imarzo@unizar.es (I.M.); jnaval@unizar.es (J.N.); 2 Hematology Department, Lozano Blesa Hospital, 50009 Zaragoza, Spain; gemma@azaceta.net (G.A.); lpalomera@salud.aragon.es (L.P.); 3 Institut of Regenerative Medicine and Biotherapy, University of Montpellier, INSERM, CNRS, University Hospital Center Montpellier, 34000 Montpellier, France; martin.villalba@inserm.fr; 4 Institut Sainte-Catherine, 84918 Avignon, France

**Keywords:** NK cells, multiple myeloma, daratumumab, isatuximab, autologous, allogeneic

## Abstract

Natural killer (NK) cell-based therapies have emerged as promising anticancer treatments due to their potency as cytolytic effectors and synergy with concurrent treatments. Multiple myeloma (MM) is an aggressive B-cell malignancy that, despite development of novel therapeutic agents, remains incurable with a high rate of relapse. In MM, the inhospitable tumor microenvironment prevents host NK cells from exerting their cytolytic function. The development of NK cell immunotherapy works to overcome this altered immune landscape and can be classified in two major groups based on the origin of the cell: autologous or allogeneic. In this review, we compare the treatments in each group, such as autologous chimeric antigen receptor (CAR) NKs and allogeneic off-the-shelf NK cell infusions, and their combinatorial effect with existing MM therapies including monoclonal antibodies and proteasome inhibitors. We also discuss their placement in clinical treatment regimens based on the immune profile of each patient. Through this examination, we would like to discover precisely when each NK cell-based treatment will produce the maximum benefit to the MM patient.

## 1. Introduction

From Dr. William B. Coley’s first foray with bacterial injections into tumors in the 1890s to the 2018 Nobel Prize in Physiology or Medicine being awarded to Jim Allison and Tasuku Honjo for immune checkpoint inhibitors, immunotherapy has always looked towards being a cancer treatment. Immunotherapeutic approaches have focused on harnessing the adaptive immune response. Here we focus on Natural Killer (NK) cells, powerful members of the innate lymphoid cell family [1] that possess features of “adaptive” or rather, trained immunity [2]. They respond rapidly, without antigen specificity, during cellular transformation or viral infection. As innate lymphoid cells, NK cells target tumor cells through direct target killing and by the release of inflammatory cytokines. 

### 1.1. NK Cell Function

With their discovery in the early 1970s, NK cells were thought to be specialized cells geared towards eliminating cancer cells. Specifically, they were described as small granular lymphoid cells that exerted a cytotoxic function against leukemia cells [3,4]. Once it was identified that NK cells were not of the monocyte or T cell lineage, their function and biology became easier to clarify [5]. As opposed to other cells also originating from the common lymphoid progenitor, NK cells do not require prior sensitization to attain cytolytic activity. While they develop in non-nodal sites, such as the bone marrow and liver, they comprise 10–15% of total lymphocytes found in peripheral blood. NK cells constitute an ideal adoptive transfer treatment because of their multifaceted cytolytic biology and diverse mechanisms for activation. Their main function is antiviral, especially against viruses that induce loss of MHC-I expression, such as the herpes virus family. Accordingly, patients with impaired NK cell function are prone to viral infection [6]. Their role in immune surveillance against tumors is also well established [1,7,8].

### 1.2. Major Subsets of NK Cells

As NK cells express the NCAM-1 molecule, which clusters as CD56, they are identified as CD56^+^CD3^−^ lymphocytes. The difference in CD56 expression intensity divides NK cells into two major subsets: CD56^bright^ and CD56^dim^ cells, both types with unique functions and capabilities [9]. CD56^bright^ cells produce pro-inflammatory cytokines, have a low expression of killer immunoglobulin-like receptors (KIR) and show a low level of cytotoxic activity. CD56^dim^ NK cells constitute the majority of NK cells in peripheral blood and express greater amounts of CD16 on their cell surface than their bright counterpart. CD56^dim^ NK cells are cytotoxic and are also able to induce potent antibody-dependent cellular cytotoxicity (ADCC) through CD16 binding to the Fc fraction of IgG [10]. In lymph nodes, CD56^bright^ cells are the most common population and NK cell development probably follows the path: CD56^brigth^CD16^−^ to CD56^brigth^CD16^dim^ to CD56^dim^CD16^dim^ to CD56^dim^CD16^+^ [11].

### 1.3. NK Cell Activation

In order to carry out any cytotoxic effect, NK cells must first discriminate between target and healthy cells. NK cells have transmembrane receptors known as KIRs that recognize HLA-I haplotypes [12], and that are able to inhibit or activate NK cell function. The KIRs containing immunoreceptor tyrosine-based inhibitory motifs (ITIMs) recruit tyrosine phosphatases and inhibit cell function. KIR haplotypes are classified into groups A and B. Haplotypes in Group A encode only inhibitory receptors with a fixed number of genes, KIR2DS4 being the only exception. Haplotypes in Group B have variable types of KIR, also including genes encoding for activating receptors [13,14]. As per Ljunggren and Karre’s “missing-self” hypothesis, NK cells select and target cells that do not express MHC-I [15]. This would allow NK cells to target only those cells “stressed” by viral infection or by neoplastic transformation and not healthy self-tissue. Another important inhibitory NK cell receptor is the C-type lectin-like heterodimeric receptor NKG2A and its isoform NKG2B, which recognize the non-classical MHC class I molecule HLA-E [16,17].

On the other hand, NK cells express numerous and variable activating receptors, which recognize viral determinants and/or stress-induced cellular ligands. Between them, natural cytotoxicity receptors (NCR) were one of the first families of activating receptors to be described. This family includes NKp46, NKp30 and NKp44, with NKp46 and NKp30 being constitutively expressed while NKp44 is only expressed after NK cell activation. NCR ligands include influenza virus hemagglutinin for NKp46, B7-H6 for NKp30 [1] and a tumor-associated ligand for NKp44 [18]. Another activating receptor is NKG2D, a C-type lectin-like receptor, which is constitutively expressed as a homodimer on the surface of NK cells. NKG2D have several types of ligands: the MHC class I chain-related molecules A and B (MICA and MICB) and the UL16-binding proteins (ULBP; [19]). Other NK cell activating receptors are the heterodimeric receptor CD94/NKG2C, which interacts with HLA-E [20], and DNAM-1, which compete with TIGIT and CD96 to bind to its ligands, CD155 and CD112 [21].

NK cell activation depends on signals mediated by activating and inhibitory receptors, the final functional outcome being the result of the balance between those activating and inhibitory signals [22]. It is remarkable that in a hematopoietic transplant setting, unlike T cells, NK cells do not elicit graft versus host disease (GvHD; [23]). However, the missing-self theory predicts that donor NK cells should react against recipient cells that do not express the HLA molecules on which donor NK cells have been educated. The experimental result means that NK cells do not attack healthy, non-stressed cells which do not express ligands for their activating receptors, although NK cells were not receiving the KIR-mediated inhibitory signals. On the contrary, as indicated below, donor NK cells are able to attack recipient leukemic cells, since in this case, in addition to the absence of inhibitory signals, tumor cells provide ligands for donor NK cell activating receptors.

Between the activating NK cell receptors, CD16 is particularly important as it mediates potent ADCC. This Fc receptor is crucial for the anti-tumor activity of therapeutic monoclonal antibodies normally directed against molecules over-expressed by tumor cells [24].

## 2. Killing of Tumor Cells by NK Cells

Once a target cell has been identified and the correct NK cell activating receptors have been engaged, granule exocytosis is activated inside the NK cell. Granules containing perforin and granzymes are released with perforin creating a pore in the membrane of the target cell [25]. Both perforin [26] and at least granzyme A and B [27] are needed for tumor cell killing by NK cells. Granzymes enter the cytosol of the tumor target cell through the perforin pore and are able to induce several types of cell death, including apoptosis, necroptosis or pyroptosis, depending on the tumor target [28]. The presence of Fas ligand (FasL) and TNF-related apoptosis inducing ligand (TRAIL) on the surface of NK cells also provide secondary pathways through which to exercise their lytic actions [25]. NK cells have been shown to exert cytotoxicity through FasL expression [29], but it seems that TRAIL is more relevant for NK cell cytotoxicity [30]. NK cells also carry out anti-tumor activities through the production of IFN-gamma. This pleiotropic cytokine is able to induce apoptosis in some circumstances, but also to inhibit angiogenesis and contribute to the activation of both innate and adaptive immune anti-tumor responses [31].

Frequently, tumor cells, and especially metastatic tumor cells, reduce their MHC-I expression, allowing them to escape from recognition by cytotoxic T cells (CTL) and from immune surveillance [32]. In fact, mutations in β2-microglobulin that result in the impairment of MHC-I expression, are associated with resistance to anti-tumor CTL and the generation of evading lesions [33] and also with resistance to immune checkpoint inhibitor immunotherapy [34]. In this case, those tumor cells should be more sensitive to NK cell-mediated elimination, making NK-cell adoptive cell transfer a therapy of choice.

In addition, and in connection with the absence of GvHD mediation by NK cells in hematopoietic transplants, NK alloreactivity can also be exploited for the treatment of hematological cancers. This has been clearly demonstrated in pioneer studies that exploited NK cell alloreactivity for the treatment of blood-borne cancers [35,36,37]. Remarkably, it has been shown, at least on ex vivo cells from B-CLL patients, that KIR mismatch is not relevant when properly expanded and activated NK cells are used as effectors [38].

Phase I/II clinical studies have been performed using NK cells expanded through different approaches on multiple myeloma and acute myeloid leukemia (AML) including pediatric patients [39,40,41,42].

We will look at the dysfunctional aspects of the MM microenvironment that cause NK cell dysfunction and the clinical trials using autologous or allogeneic NK cell therapies to overcome these immunogenic hurdles.

## 3. NK Cells in Multiple Myeloma

### 3.1. Current Therapies for Multiple Myeloma

Multiple myeloma (MM) is the second most common hematological malignancy, characterized by the clonal expansion of plasma cells in the bone marrow [43,44]. Once a disorder without effective treatment, over the past two decades, new treatments such as autologous stem cell transplant (ASCT), immunomodulatory drugs (IMiDs), proteasome inhibitors and monoclonal antibodies have improved the survival rates of myeloma patients [45]. Current median survival is 6 years, and relapse, even after complete remission, is very common [45,46,47]. Regarding IMiDs, preclinical data showed that lenalidomide enhances anti-myeloma cellular immunity mediated by CD8^+^ T cells and by NK cells [48]. Later on, it was described that lenalidomide was able to reduce the expression of PD-1 in CD8^+^ T lymphocytes and in NK cells, and of PD-L1 in MM cells and bone marrow accessory cells [49]. The combination of expanded NK cells with proteasome inhibitors has also been demonstrated to increase their cytotoxic potential [50]. Currently, through the identification of tumor cell surface markers, e.g., CD38 [51], the development of targeted antibodies and adoptive cell therapies have been developed. While many of the available therapies induce remission in new onset cases, relapse, often with acquired resistance, occurs [52]. There is thus a persistent need for novel and combinatorial therapies. As it will be indicated below in the description of the NK cell-based clinical assays, the combination with proteasome inhibitors and especially with IMiDs is given the best clinical results.

### 3.2. Antibody-Based Therapy of Multiple Myeloma

Antibody-based therapies rely on unique or over-expressed proteins on the surface of aberrant cells. In MM, the cell surface single-chain transmembrane glycoprotein CD38 is highly expressed and used as part of the definitive phenotype for MM cells. As MM cells have a high surface density of CD38, it has become the target for antibody therapy [53]. Daratumumab, a fully humanized IgG1 κ mAb, was the first to target CD38 and gain approval for MM treatment. Daratumumab has been approved as both monotherapy and in combination with several regimens of proteasome inhibitors and chemotherapeutic agents [54,55]. It causes the death of myeloma cells primarily through ADCC. This process occurs through the crosslinking of CD38-bound antibody on MM cells by the CD16 receptors on NK cells. Other pathways of action include antibody-dependent cellular phagocytosis (ADCP) and complement-dependent cytotoxicity (CDC, [56]). Of note, NK cells also express CD38 on their surface, albeit at a lower level than myeloma cells. It has been reported that daratumumab causes fratricide between NK cells [57,58]. However, CD38 expression on myeloma or NK cells can be balanced to favor myeloma killing by daratumumab [59,60]. In addition, it has been demonstrated using two different expansion protocols that the deleterious effect of daratumumab is reduced on properly activated and expanded NK cells, demonstrating that the potential of the combination therapy is stronger than the possible fratricidal side effects [57,61]. Daratumumab augments, at least in an ex vivo model, the dysfunctional NK cell activity caused by the hypoxic and acidified MM microenvironment [62].

Isatuximab is a novel chimeric mouse/human IgG1κ mAb that also targets CD38. It is currently approved in combination with pomalidomide/dexamethasone in relapse/refractory patients with at least two prior therapies [63]. Although directed at the same target as daratumumab, it is principally dependent on the ADCC pathway and less on CDC [64]. There are several ongoing clinical trials examining its effectiveness alone and in combination with classic chemotherapies and proteasome inhibitors [65].

Elotuzumab is a humanized immunoglobulin G1 immunostimulatory mAb that targets the signaling lymphocytic activation molecule family member 7 (SLAMF7; [66]). This mAb is currently approved for relapsed and refractory MM [67]. SLAMF7 is expressed on both NK and myeloma cells and exerts its effect by activating NK cells directly and also by mediating CD16-dependent ADCC [68].

The use of mAbs has greatly improved treatment outcomes in MM, however, patients continue to relapse. Part of this is due to the heavy reliance of mAbs on functional NK cells to mediate ADCC. The suppressive MM microenvironment actively inhibits the function of immune cells, including NK cells. To overcome drug resistance and improve long-term treatment outcomes, the MM microenvironment must be explored, and its deleterious effects limited.

### 3.3. MM Microenvironment. NK Cell Dysfunction

Alterations within the bone marrow microenvironment (BMM) guide the progress and ongoing persistence of MM. MM patients show variable infiltration of immune cells, even in the early disease stage [69]. Remarkable changes in immune cell populations begin during precursor stages of MM, particularly MGUS [45]. The BMM contains NK cells, T and B-lymphocytes, a balance of osteoclasts and osteoblasts, fibroblasts, bone marrow stromal cells, endothelial cells, the extracellular matrix and blood vessels. Important growth factors, chemokines and cytokines are secreted by the stroma. Progressive immune deregulation impairs T, B, APCs and NK cell function in the MM niche [70]. Deficits in the humoral immune response are common in MM due to a reduction of bone marrow B-cell progenitors [71]. An immunosuppressive microenvironment is generated through the increased infiltration of regulatory T cells (Tregs), generating also aberrant CD4/CD8 ratios [72,73]. Additionally, an expansion of the myeloid-derived suppressor cells (MDSCs) population correlates with disease progression and with negative patient outcome [74]. On the other hand, BM stromal cells (BMSCs) favor myeloma cell proliferation and survival by cell-to-cell contacts and by the secretion of soluble factors [75]. Finally, several studies have shown impaired dendritic cell function in the MM microenvironment [76,77]. Genetic abnormalities in myeloma cells deregulate signaling and transport pathways and increase the expression of anti-apoptotic molecules [78], favoring their escape from immune surveillance as disease progresses [79]. Expression of immune checkpoint inhibitors is believed to be a mechanism of tumor escape in multiple types of cancers, including MM. Increased expression of PD-L1 on MM cells, combined with PD-1 expression on T cells are indicators of poor prognosis [80,81].

Consequently, the anti-myeloma effect of NK cells is be tempered by the immunosuppressive MM tumor microenvironment that leads to dysfunctional cytotoxicity. Despite this, MM patients have NK cells that are actively interacting and killing MM cells [82,83]. In fact, while Garcia-Sanz et al. reported an increase in NK cells found in the peripheral blood and bone marrow of MM patients, other studies detected a decrease in overall NK cell numbers [84,85]. On the other hand, of all lymphocyte subsets, only NK cell numbers increased after autologous stem cell transplantation in patients with long-term disease [86]. Total numbers aside, NK cells present in the tumor microenvironment are impaired and their activation and function decline as disease-stage progresses [87]. Indeed, the changes undergone by NK cells in the BMM have been implicated in the progression of MGUS to MM [88].

As previously discussed, NK cells require activating signals such as those provided by NCRs, NKG2D or DNAM-1 ligation in order to lyse target cells. The ligand for NKG2D most abundantly expressed by MM cells is MICA. As disease progresses, MICA is shed from the surface of MM cells and NKG2D is internalized, avoiding NK cell activation by this pathway [89,90]. DNAM-1 expression is also reduced as MM progresses while its ligand PVR is upregulated. Likewise, SLAMF7 and the FcyRIII CD16 have lower expression levels than in healthy controls [91].

In conjunction with the increase in PD-L1 expression on MM cells, NK cells also present up-regulation in the expression of PD-1 [92]. While the PD-1/PD-L1 axis is a promising avenue of treatment in most cancers, clinical assays in MM combining those checkpoint inhibitors and IMiDs have been underwhelming and have been discontinued (see the NTC02579863, NTC02576977, NCT02726581 and NCT02431208 clinical assays [93,94]). However, a recent study combining pembrolizumab with lenalidomide, dexamethasone and autologous transplant gave good results [95]. Presence of PD-1 on NK cells, even when the axis is blocked through the use of immune checkpoint inhibitors, do not result in increased cytolytic NK cell activity. Indeed, expression of PD-1 on NK cells appears to be a marker of exhaustion [61,96].

The most abundant cytokines in the MM microenvironment are IL-6 and IL-10, secreted primarily by perivascular cells and Tregs, respectively [97,98]. While IL-6 has both pro- and anti-inflammatory roles, in MM it exerts an immunosuppressive effect on NK cells and CTLs. In NK cells, IL-6 initiates a signal transduction pathway that implicates NF-κB and STAT3, resulting in reduced perforin expression and other down-regulations of NK cell cytotoxicity [99,100]. IL-6 also stimulates tumor cell proliferation, metastasis and survival [101]. Both MM cells and Tregs infiltrated in the MM microenvironment secrete the immunosuppressive cytokine TGF-β [102].

**Figure 1:**
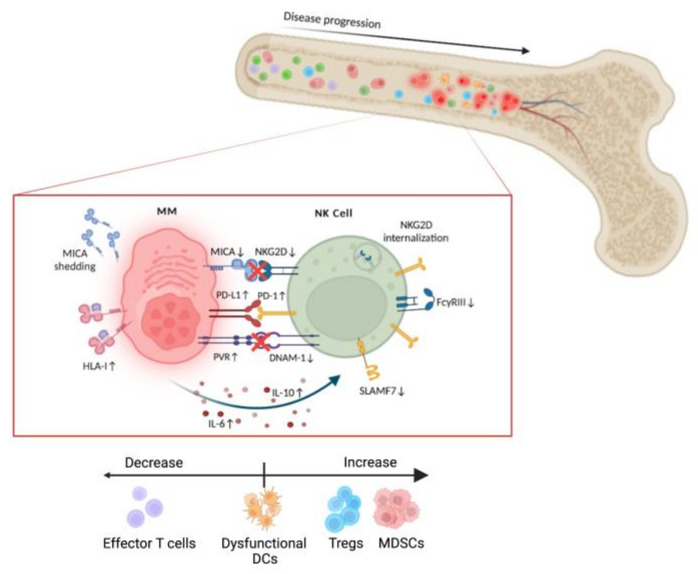
Schematic representation of the immunosuppressive MM microenvironment and the deleterious effect on NK cell anti-myeloma function. Created with BioRender.com.

The most important effects of the MM tumor microenvironment on the inhibition of NK cell function are shown in Figure 1.

Due to this extremely unfavorable microenvironment, patient NK cells frequently lack the robustness necessary to have an impact on MM development. In consequence, adoptive treatment with functionally activated NK cells will be key to augmenting the abilities of existing MM therapies. To this effect, there are several ongoing clinical trials that explore this immune niche, which are going to be described in the next section.

## 4. NK Cell-Based Treatment for MM

In order to overcome the above-described NK-cell dysregulation, several clinical studies have and are being carried out with NK cells as adoptive cell transfer therapies in MM patients (see also the recent review by Liu et al. [103]). There are 22 clinical trials registered in the clinicaltrials.gov database that use NK cells for the treatment of MM, alone or in combination with other therapies. The studies can be stratified based on the source of the NK cells used, whether they are autologous or allogeneic as described in Table 1 and Table 2, respectively. In order to reach their full potential, NK cells should be activated and expanded ex vivo or supported through the addition of cytokines alongside NK cell infusions. A variety of “feeder” cells engineered to express ligands that activate the NK cells are used along with cytokines such as IL-2 and IL-15 for the maintenance and expansion of healthy, cytotoxic NK cells. The utilization of NK cells in adoptive cell transfer therapy was first trialed in 1985 on patients with metastatic cancers. The results showed no long-term clinical benefit, but NK cells were detected in the patients’ fluids weeks after the infusions [104]. More recent NK cell-based trials have tried to capitalize on NK cell persistence while improving clinical results.

### 4.1. Autologous NK Cells

Autologous SCT after induction therapy for MM remains the standard of care for patients who are transplant eligible. While effective in temporarily holding the disease at bay, it is not curative. The addition of autologous cell transfer therapies can help prolong the effect of ASCT [105].

Of the clinical trials presented in Table 1, the team of Dr. Frits van Rhee at the University of Arkansas has led three: NCT01884688, NCT01313897 and NCT03003728. The two first assays showed that NK cells from heavily pre-treated MM patients could be expanded using K562-mbIL15- 41BBL feeder cells and that the combination with bortezomib, a proteasome inhibitor, before the NK cell infusion improved the results. Of the 10 patients enrolled in NCT01313897, 5 used autologous NK cells and 3 used haploidentical NK cells. Two patients were able to reach 6 months without further therapy (1 PR, 1 decrease in disease progression). The other 5 patients did not see a change in disease progression [42]. NCT03003728, which used eNK cells combined with elotuzumab, was withdrawn without definitive results.

The FDA recently gave orphan drug status to CellProtect, an autologous NK cell product that is frozen and delivered to clinics (NCT04558853). This product was trialed at Karolinska University hospital in newly diagnosed MM patients who had undergone ASCT. No severe adverse events were reported and of the 6 participants, 4 continued to have measurable disease after ASCT but then responded to NK cell treatment. The clinical trial NCT04558931 using this same CellProtect product with the addition of isatuximab in newly diagnosed MM patients is under the recruitment phase at this moment.

The combination of autologous NK cells and bortezomib was also studied in two further trials. NCT00720785 consisted of 35 participants with a variety of cancers including multiple myeloma. Each patient was treated with 3 escalating doses of ex vivo expanded NK cells. Those with hematological malignancies were sensitized to NK cell cytotoxicity through the addition of bortezomib. In NCT02481934, patients were stratified into two arms, 3 patients received lenalidomide while 2 received bortezomib. After infusions, there was an increase in NK cells and in the expression of its activating receptors NKp30 and NKG2D [40]. One patient was able to maintain partial response for 13 months after infusion. Of the others who completed the NK treatment, each achieved disease stabilization, which persisted for at least 4 months before disease progression. The only patient that was unable to finish the NK infusion due to unrelated toxicity experienced disease progression after 2 months, indicating the need to receive the full dose of NK cells.

The completed studies clearly show that autologous activated NK cells have anti-myeloma activity and that infusions from patient NK cells are possible.

### 4.2. Allogeneic NK Cells

Allogeneic NK cells are a convenient option that does not rely on the viability of patient NK cells. As each patient presents with a different treatment history and unique set of markers, expansion of autologous cells can be difficult and may fail. Induction of remission in patients with advanced acute leukemia was shown in pioneer clinical trials using allogeneic NK cells [106]. All studies are necessarily based on patients that have undergone some form of stem cell transplant. The success of lymphocyte reconstitution, particularly that of NK cells, is associated with better progression-free survival [107]. This applies to patients having undergone allogeneic hematopoietic stem cell transplantation due to the possible NK cell-mediated effects of graft-versus-tumor (GVT) [108].

To provide a more universal, off-the-shelf product, there have been several clinical trials in MM based on different forms of allogeneic NK cell therapy, summarized in Table 2. These studies are based on patients that have received allogeneic HSCT. The addition of allogeneic NK cells to augment the lymphocyte population should help in the prolongation of PFS. Several studies showed that treatment with allogeneic NK cells was well tolerated with no evidence of GvH disease (GvHD). However, relapse, progression-free survival and overall survival were not significantly different from patients only having undergone ASCT (clinical trial NCT01040026; [109]).

Not all studies are based on haploidentical-donor-derived NK cells. In NCT04309084, allogeneic CYNK-001 NK cells are used. They originate from human placental CD34^+^ cells and are enriched for the CD56^+^CD3^-^ subset after generation. These cells are infused in patients post-ASCT as a front-line treatment, in 3 dosing cohorts.

**Figure 2:**
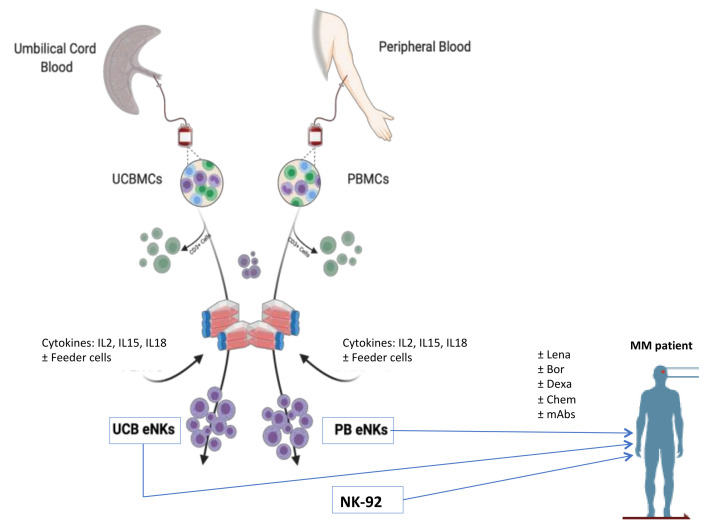
Schematic representation of the main therapeutic protocols described in Section 4.2. Abbreviations are the same as used in Table 1 and Table 2.

While many studies use adult-donor-derived NK cells, there is also an arm of clinical trials that use umbilical-cord-blood-derived NK cells (see Figure 2). In a multi-disease study out of MD Anderson, 13 patients with hematological malignancies, which included MM, were treated with ex vivo expanded umbilical cord blood NK cells (NCT01619761). The treatment plan consisted of 2 arms. In both plans, patients received a high dose of lenalidomide and fludarabine phosphate. Melphalan was added to arm 1 and cyclophosphamide plus total body irradiation (TBI) was part of arm 2. All patients received cord blood NK (CB-NK) cells prior to undergoing allogeneic umbilical cord blood transplants. The primary outcomes of this study were to generate a robust NK cell presence and to establish the treatment-related mortality. Focusing on MM, this team continued to study the safety and tolerance of CB-NKs after a high dose of melphalan and a low dose of lenalidomide (clinical trial NCT01729091; [110]). No toxicities resulted from CB-NK infusions. Of the 12 participants, 10 achieved a partial response or better. At the 21-month follow-up, only 4 patients had progressed, 2 having expired. Phase II continued with CB-NKs being used at doses of 1 × 10^8^ CB-NK/kg, employing the previous treatment scheme. Three additional studies using this therapeutic approach are in the recruiting phase: NCT02727803, NCT03019666 and NCT04754100.

Another category of allogeneic NK cells is that based on cell lines. In the phase I trial NCT00990717, NK cells from the NK-92 cell line are irradiated and given to patients in 3 escalating doses in 6 cycles [111]. Treatment resulted in minimal toxicity and was deemed safe. While 8 patients received only a fraction of the cycle due to disease progression, 3 patients did receive the full 6 cycles. Eventually patients did relapse, but the tolerability of NK-92 cells was established. Further modifications to this product are ongoing. A schematic representation of the different therapeutic protocols described is shown in Figure 2.

Overall, the clinical trials described here establish the safety and dose limitations of adoptive cell therapies with both autologous and allogeneic NK cells. Due to the variety of allogeneic products available, their ease of manipulation and off-the-shelf formatting, trials are focusing on the development of this product. Further studies need to be done to ameliorate the long-term therapeutic outcomes of these treatments.

## 5. Future Prospects

The future of cancer immunotherapy, particularly that of harnessing the cytolytic power of NK cells is wide open. As summarized above, there are several avenues being explored for effective future treatments in MM. In order to fully realize the potential of NK cell therapies, we must have strategies for addressing challenges. Beginning with NK cell functionality, there is a large range of NK cell types being tested.

For example, in [61] results showed how expanded NK cells from umbilical cord blood were highly cytotoxic against MM patient bone marrow aspirates without the addition of monoclonal antibodies. However, expanded NK cells derived from healthy human peripheral blood did have a synergistic effect when combined with daratumumab. Each type of expanded NK cells would serve well in a situation based on possible treatment combinations and patient history.

While personalized treatments such as autologous CAR NKs or CAR T cells have their place in immunotherapy, the goal should be to make viable NK cell therapies universally available in all markets. For this, allogeneic NK cells would help MM cell therapy, as they do not elicit GvHD. This therapy would be scalable causing a decrease in overall cost and would be ready off-the-shelf. Having the treatment ready for the patient immediately would greatly reduce the waiting times caused by the production of autologous cell therapies. Collaborations between the private and public sector will also make this a competitive, highly available option. Combining NK cellular therapy with existing approved immunotherapies such as mAbs is also a promising avenue. In this sense, it is important that grafted activated NK cells maintain CD16 expression to mediate ADCC [24]. As we add to the number of successful existing therapies under the pillar of cancer immunotherapy, the possible combinations available to patients in every progressing situation also increases. Personalized medicine will venture beyond only the use of autologous cells and respond to fluctuations in the patients’ genetic, phenotypic profile, and provide cellular treatments geared towards those changes. While there is still no definitive cure for multiple myeloma, the treatment landscape continues to evolve, and NK-cell-based therapy is on the horizon as an exciting, viable option that will create better outcomes for future patients.

## Figures and Tables

**Table 1:** Clinical trials based on autologous NK cell infusions. Bor—bortezomib, Elo—elotuzumab, Mel—melphalan, Len—lenalidomide, Isa—isatuximab, NR—not recruiting, * MM indicates that the trial was done in a variety of tumor types and that the number of MM patients was not specified.

Trial ID.	Specific NK Cell Source	Additional Treatment	Cytokine Support	Phase	Status (# Patients)	Trial Title
**NCT01884688**	K562-mb15-41BBL	-	IL-2	II	Completed (1)	A Phase II Study of Autologous Expanded Natural Killer Cell Therapy for Asymptomatic Multiple Myeloma
**NCT01313897**	K562-mb15-41BBL	Bor	IL-2	II	Completed (10)	UARK 2010-35, A Phase II Study of Expanded Natural Killer Cell Therapy for Multiple Myeloma
**NCT03003728**	K562-mb15-41BBL	Elo, Mel	IL-15 (ALT-803)	I	Withdrawn (10)	2015-10: A Phase II Pilot Study of Expanded Natural Killer Cells and Elotuzumab to Eradicate High-Risk Myeloma Post Autologous Stem Cell Transplant
**NCT04558853**	Autologous	-	-	I/II	Active, NR (6)	A Safety Study of CellProtect, an Autologous ex Vivo Expanded and Activated Natural Killer (NK) Cell Product, in Patients with Multiple Myeloma
**NCT00720785**	Autologous	Bor	-	I	Completed (35, * MM)	Safety and the Anti- Tumor Effects of Escalating Doses of Adoptively Infused Ex Vivo Expanded Autologous Natural Killer (NK) Cells Against Metastatic Cancers or Hematological Malignancies Sensitized to NK-TRAIL Cytotoxicity with Bortezomib
**NCT02481934**	K562-mb15-41BBL	Len, Bor	-	I	Completed (5)	Phase 1 Clinical Trial to Evaluate Security and Dose of Expanded and Activated Autologous NK Cells Infusions in Consolidation of Multiple Myeloma Patients Treatment on Second or Later Relapse
**NCT04558931**	Autologous	Isa	-	II	Recruiting	An Open, Randomized, Controlled, Phase II Trial of CellProtect in Combination with Isatuximab Antibody Versus Isatuximab Antibody Alone as Maintenance Treatment in Patients with Multiple Myeloma Undergoing High Dose Treatment

**Table 2:** Clinical trials based on allogenic NK cell infusions. Dex—dexamethasone, Cyc—cylophosphamide, Mel—melphalan, Flu—fludarabine, Bor—bortezomib, Elo—elotuzumab, Len—lenalidomide, Myc—mycophenolate mofetil, Rit—rituximab, ATG—anti-thymocyte globulin, Bus—busulfan, Clo—clofarabine, NR—not recruiting, NRP—no results posted, * MM indicates that the trial was done in a variety of tumor types and that the number of MM patients was not specified.

Trial ID	Specific NK Cell Source	Additional Treatment	Cytokine Support	Phase	Status (# Patients)	Trial Title
**NCT0008945**	KIR-L mismatch haploidentical	Dex, Cyc, Mel, Flu, Bor	IL-2	I/II	Completed, NRP (10)	UARK 2003-18, A Phase II Study of KIR-Ligand Mismatched Haplo-Identical Natural Killer Cells Transfused Before Autologous Stem Cell Transplant in Relapsed Multiple Myeloma
**NCT00569283**	Allogenic	-	-	I	Completed, NRP (18, * MM)	Donor Natural Killer Cell Infusion for the Prevention of Relapse or Graft Failure After HLA-Haploidentical Familial Donor Bone Marrow Transplantation-A Phase I Study
**NCT00660166**	HLA Class I Haplotype Mismatched	Ben	-	I	Completed, NRP (13, * MM)	HLA Class I Haplotype Mismatched Natural Killer Cell Infusions After Autologous Stem Cell Transplant for Hematological Malignancies
**NCT00789776**	Allogenic	-	-	I/II	Completed (41, * MM)	A Phase I/II Study Evaluating the Safety and Efficacy of Adding a Single Prophylactic Donor Lymphocyte Infusion (DLI) of Natural Killer Cells Early After Nonmyeloablative, HLA-Haploidentical Hematopoietic Cell Transplantation—A Multi-Center Trial
**NCT00823524**	Allogenic	-	-	I/II	Completed (47, * MM)	Donor NK Cell Infusion for Progression/Recurrence of Underlying Malignant Disorders After HLA-haploidentical HCT—a Phase 1-2 Study
**NCT00990717**	NK-92 cells	-	-	I	Completed (11)	A Dose Escalation Study of NK-92 Cell Infusions in Patients with Hematological Malignancies in Relapse After Autologous Stem Cell Transplantation
**NCT02955550**	PNK-007	Mel	rhIL-2	I	Completed (15)	A Phase 1, Multicenter, Open-label, Safety Study of Human Cord Blood Derived, Culture-expanded, Natural Killer Cell (PNK-007) Infusion Following Autologous Stem Cell Transplant for Multiple Myeloma
**NCT01040026**	Haploidentical	Mel	-	I/II	Active, NR (10)	A Phase I/II Single Center Study to Assess Tolerability and Feasibility of Infusions of Allogeneic Expanded Haploidentical Natural Killer (NK) Cells in Patients Treated with High Dose Melphalan Chemotherapy and Autologous Stem Cell Transplantation for a Multiple Myeloma
**NCT04309084**	CYNK-001	-	-	I	Active, NR (29, * MM)	A Phase I Study of Human Placental Hematopoietic Stem Cell Derived Natural Killer Cells (CYNK 001) in Multiple Myeloma Patients Following Autologous Stem Cell Transplant in the Front-line Setting
**NCT01619761**	UCB	Mel, Len, Flu, Myc, Cyc, Rit	-	I	Active, NR (12)	Natural Killer Cells in Allogeneic Cord Blood Transplantation
**NCT01729091**	UCB	Elo, Len, Mel	-	II	Active, NR (72)	Phase II Study of Umbilical Cord Blood-Derived Natural Killer Cells in Conjunction with Elotuzumab, Lenalidomide and High Dose Melphalan Followed by Autologous Stem Cell Transplant for Patients With Multiple Myeloma
**NCT02890758**	Non-HLA matched donor	-	ALT803	I	Active, NR (14, * MM)	Phase I Trial of Universal Donor NK Cell Therapy in Combination With ALT-803
**NCT02727803**	NK-92	ATG, Flu, Cyc, Clo, Bus	-	II	Recruiting	Personalized NK Cell Therapy in Cord Blood Transplantation
**NCT03019666**	NAM-NK	Cyc, Flu	-	I	Recruiting	A Phase I Trial Testing NAM Expanded Haploidentical or Mismatched Related Donor Natural Killer (NK) Cells Followed by a Short Course of IL-2 for the Treatment of Relapsed/Refractory Multiple Myeloma and Relapsed/Refractory CD20+ Non-Hodgkin Lymphoma
**NCT04754100**	agent-797	-	-	I	Recruiting	A Phase I Open-Label Study of the Safety, Tolerability and Preliminary Clinical Activity of Allogeneic Invariant Natural Killer (iNKT) Non-transduced Cells (agenT-797) in Patients with Relapsed/Refractory Multiple Myeloma

## Data Availability

Not applicable.
